# Oroxylin A Inhibits Hemolysis via Hindering the Self-Assembly of α-Hemolysin Heptameric Transmembrane Pore

**DOI:** 10.1371/journal.pcbi.1002869

**Published:** 2013-01-17

**Authors:** Jing Dong, Jiazhang Qiu, Yu Zhang, Chongjian Lu, Xiaohan Dai, Jianfeng Wang, Hongen Li, Xin Wang, Wei Tan, Mingjing Luo, Xiaodi Niu, Xuming Deng

**Affiliations:** 1 Key Laboratory of Zoonosis, Ministry of Education, Institute of Zoonosis, College of Veterinary Medicine, Jilin University, Changchun, China; 2 Department of Food Quality and Safety, Jilin University, Changchun, China; UNC Charlotte, United States of America

## Abstract

Alpha-hemolysin (α-HL) is a self-assembling, channel-forming toxin produced by most *Staphylococcus aureus* strains as a 33.2-kDa soluble monomer. Upon binding to a susceptible cell membrane, the monomer self-assembles to form a 232.4-kDa heptamer that ultimately causes host cell lysis and death. Consequently, α-HL plays a significant role in the pathogenesis of *S. aureus* infections, such as pneumonia, mastitis, keratitis and arthritis. In this paper, experimental studies show that oroxylin A (ORO), a natural compound without anti-*S. aureus* activity, can inhibit the hemolytic activity of α-HL. Molecular dynamics simulations, free energy calculations, and mutagenesis assays were performed to understand the formation of the α-HL-ORO complex. This combined approach revealed that the catalytic mechanism of inhibition involves the direct binding of ORO to α-HL, which blocks the conformational transition of the critical “Loop” region of the α-HL protein thereby inhibiting its hemolytic activity. This mechanism was confirmed by experimental data obtained from a deoxycholate-induced oligomerization assay. It was also found that, in a co-culture system with *S. aureus* and human alveolar epithelial (A549) cells, ORO could protect against α-HL-mediated injury. These findings indicate that ORO hinders the lytic activity of α-HL through a novel mechanism, which should facilitate the design of new and more effective antibacterial agents against *S. aureus*.

## Author Summary

The mechanism controlling protein-ligand interactions is one of the most important processes in rational drug design. X-ray crystallography is a traditional tool used to investigate the interaction of ligands and proteins in a complex. However, protein crystallography is inefficient, and the development of crystal technology and research remains unequally distributed. Thus, it seems impractical to explore the structure of the α-hemolysin-ORO monomer complex by crystallography. Therefore, we used molecular dynamics simulations to investigate the receptor-ligand interaction in the α-HL-ORO monomer complex. In this study, we found that oroxylin A (ORO), a natural compound with little anti-*S. aureus* activity, can inhibit the hemolytic activity of α-HL at low concentrations. Through molecular docking and molecular dynamics simulations, we determined the potential binding mode of the protein-ligand interaction. The data revealed that ORO directly binds to α-HL, an interaction that blacks the conformational transition of the critical “Loop” region in α-HL and thus prevents the formation of the α-HL heptameric transmembrane pore, which ultimately inhibits the hemolytic activity of α-HL. This mechanism was confirmed by experimental data. Furthermore, we demonstrated that ORO could protect against α-HL-mediated injury in human alveolar epithelial (A549) cells.

## Introduction

*Staphylococcus aureus* is an opportunistic pathogen in humans and other mammals that causes many different types of infections, including superficial abscesses, septic arthritis, osteomyelitis, pneumonia, endocarditis, and sepsis [1], [2]. The number of virulence factors secreted by *S. aureus*, including extracellular and cell wall-related proteins, determines its pathogenicity [3]. The virulence factor α-hemolysin (α-HL) is one of the most important factors produced by the majority of *S. aureus* strains and recent studies have demonstrated that it plays a major role in *S. aureus* pneumonia [4]. Previous studies using a mouse model of *S. aureus* pneumonia have shown that *S. aureus* strains that lack the *hla* gene (and thus do not secrete α-HL) cause less lung injury and inflammation than the *hla* positive strains [5].

The α-HL protein, isolated from the gram-positive pathogenic bacterium *S. aureus*, is a well-studied model that has been used to elucidate mechanisms of membrane insertion by soluble proteins. Studies have shown that α-HL can self-assemble on the lipid bilayers of the membranes of susceptible host cells to form a wide heptameric pore [6]. The protein is toxic for a wide range of mammalian cells, particularly erythrocytes and epithelial cells and serves primarily as a tool that converts host tissue into nutrients for any bacteria that expresses it [3].

In an effort to increase our understanding of the function of α-HL, the structure of the heptameric pore was resolved by X-ray crystallography to a resolution of 0.19 nm [6]. Contained within the mushroom-shaped homo-oligomeric heptamer is a 10 nm long solvent-filled channel that runs along the seven-fold axis and ranges from 1.4 nm to 4.6 nm in diameter. The lytic transmembrane domain forms the lower half of a 14-strand antiparallel β barrel, to which each protomer contributes two 6.5 nm long β strands. Considering the essential nature of the heptameric crystal structure, Ragle et al. used a modified β-cyclodextrin compound, IB201, to prevent the α-HL-induced lysis of human alveolar epithelial cells (A549) [7]. This protective effect does not result from the ability of β-cyclodextrin to impair formation of the oligomeric α-HL on the cell surface, supporting a role for this molecule in the blockade of the lytic pore. Previous investigations had demonstrated the use of unsubstituted β-cyclodextrin as an adapter molecule that is capable of lodging within the central pore of α-HL and can thus facilitate the use of the toxin as a biosensor [8], [9]. The investigation of β-cyclodextrin using IB201 revealed that it blocks ion conductance through the assembled hemolysin pore, which supports the finding that β-cyclodextrin inserts into the pore itself. Although the inhibitory effect of β-cyclodextrin on ion conductance and red blood cell hemolysis were both observed in the low micromolar concentration range, this treatment strategy is passive. It is clear that prior to inhibition by β-cyclodextrin, the oligomeric α-HL on the cell surface has been formed and the cell has been damaged. Therefore, further research to identify new potent inhibitors is essential.

In our previous study, we reported that baicalin (BAI), a natural compound could bind with α-HL directly and inhibit the hemolytic activity of by restraining the conformation change of the binding cavity, “triangle region” (residues 147–153) [10]. In this study, we found that another natural compound, oroxylin A (ORO) could inhibit the hemolytic activity of α-HL stronger. Surprisingly, based on molecular dynamics simulations and free energy calculations, a new mechanism of inhibition was obtained compared with baicalin (BAI), which is that ORO bind to new active sites (residues Thr12 and Ile14) of α-HL and inhibit the hemolytic activity of α-HL due to the binding of ORO to the critical “Loop” region of α-HL. All these results indicate that the “triangle region” (residues 147–153) is not the only active site of inhibitor bound with α-HL and “Loop” region of α-HL also plays an important role in the inhibition of hemolytic activity of α-HL. With these approaches, we identified that ORO, which binds to the active site (Thr11, Thr12, Ile14, Gly15 and Lys46) of α-HL, is a potent inhibitor of the α-HL self-assembly process. These results could provide useful in the design of novel drugs for α-HL.

## Results

### ORO inhibits the hemolytic activity of α-HL

**Figure 1:**
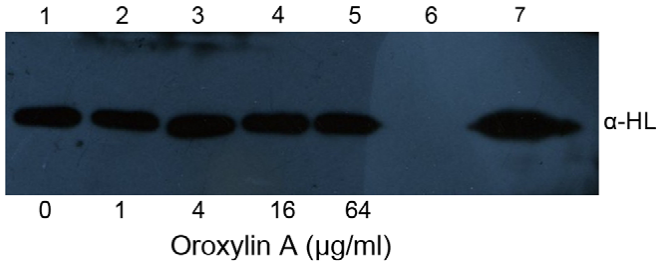
Western blot analysis of α-HL expression. Culture supernatants of *S. aureus* strains 8325-4 (lanes 1–5) and DU 1090 (lane 6) grown in increasing concentrations of ORO. Lane 7, 10 ng purified α-HL.

**Figure 2:**
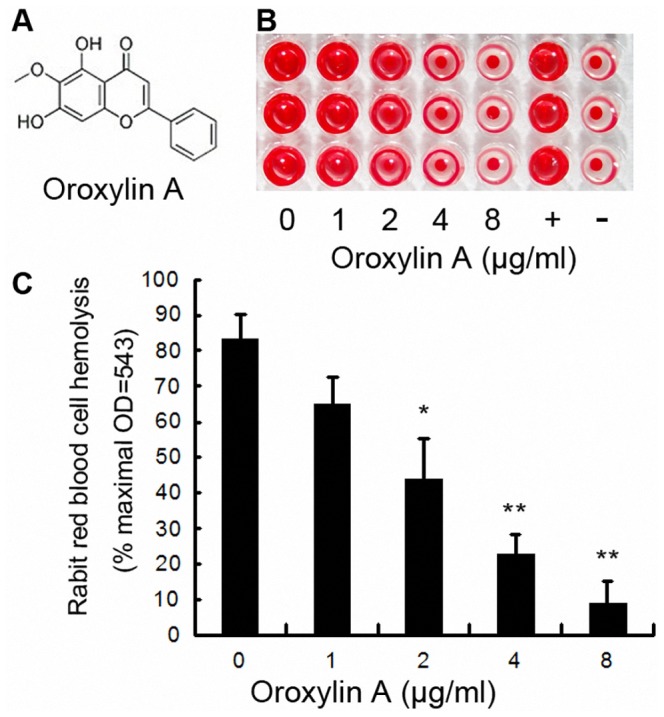
Inhibition of α-HL-induced hemolysis. A, Chemical structures of ORO; B and C, Hemolysis assays were performed with purified α-HL using rabbit red blood cells in PBS. Bars show the mean values of three independent assays (n = 3). Error bars show the standard deviations (SD). * indicates *P*<0.05 and ** indicates *P*<0.01 compared to the drug-free group.

The studies that were performed to determine the minimal inhibitory concentration (MIC) showed that the maximum concentration of ORO tested was not able to inhibit the growth of *S. aureus*, which indicates that ORO has no antimicrobial activity against *S. aureus*. Our previous study showed that many natural compounds could inhibit the hemolytic activity of the culture supernatant of *S. aureus* by decreasing the expression of α-HL [11]. In the present study, we found that ORO cannot affect the production of α-HL in *S. aureus* (Figure 1). However, ORO attenuated the hemolytic activity of purified α-HL in a concentration-dependent fashion (Figure 2B and 2C). Consequently, it is reasonable to deduce that ORO has a direct effect on α-HL.

### α-HL-ORO interactions

Based on the previous result, which showed that ORO inhibited the hemolytic activity of α-HL, we studied the binding of ORO to α-HL via molecular docking and molecular dynamics simulations using the AutoDock 4.0 and Gromacs 4.5.1 software packages, respectively. The initial structure of the monomeric α-HL was obtained from homology modeling, as previously reported [12].

**Figure 3:**
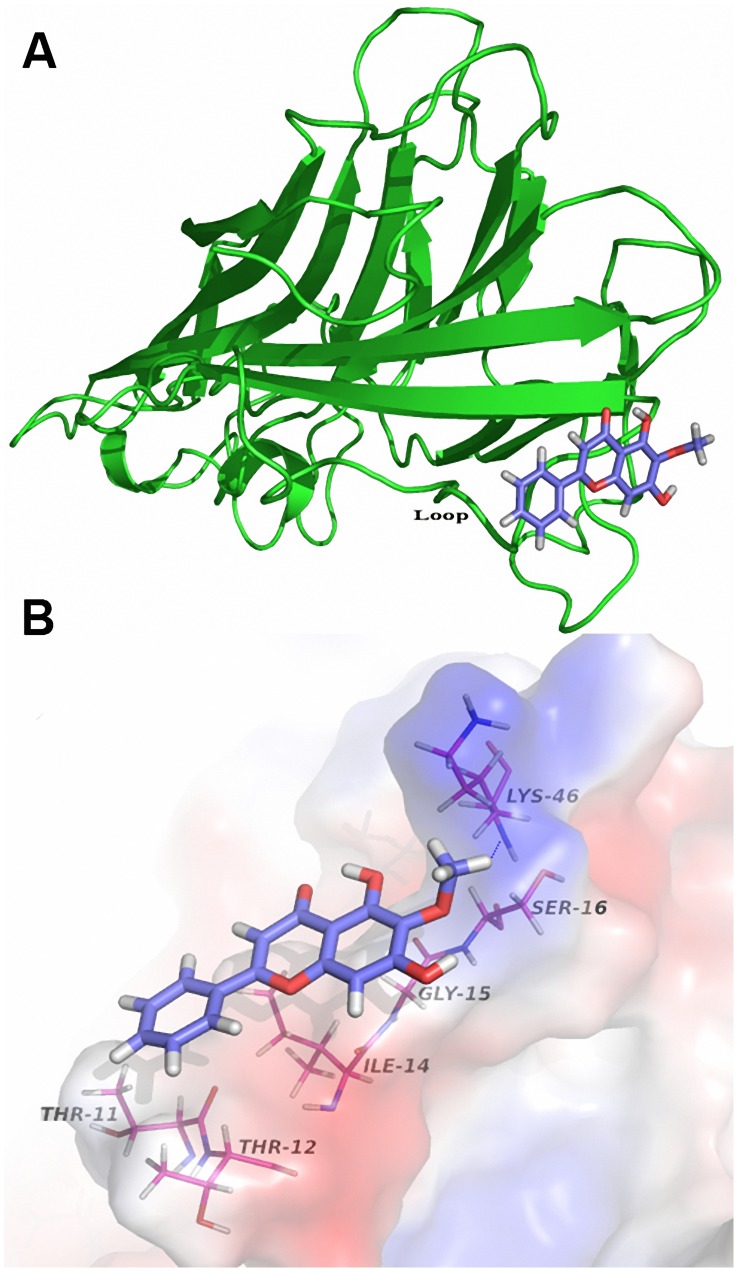
General view of the α-HL: ORO complex based on MD simulation. (A) MD simulation conformation of ORO in complex with α-HL. ORO is colored in pink. (B) Electrostatic potentials of the final binding site (blue = positive regions, red = negative regions, and white = neutral regions). Blue dotted lines indicate hydrogen bonds. The structure of the binding mode of ORO with α-HL shows the interaction of ORO with Thr11, Thr12, Ile14, Gly15 and Lys46.

**Figure 4:**
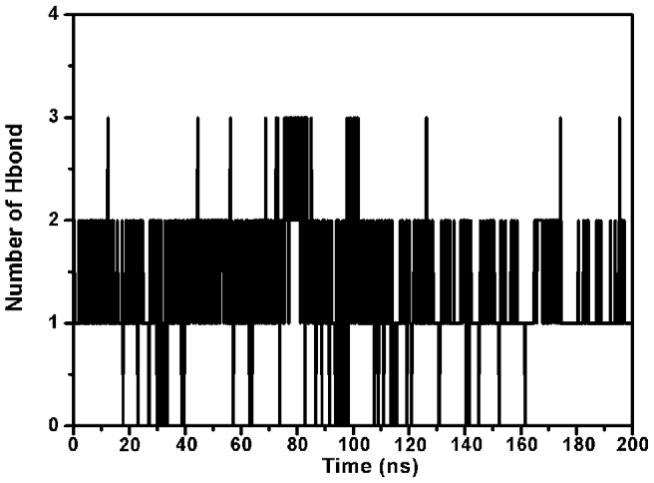
The number of H-bonds between ORO and α-HL during the 200-ns simulation. During the simulation time, the number of hydrogen bonds fluctuates mostly between 1and 2, which indicates that there is always a hydrogen bond between ORO and α-HL.

The complex structure based on the docking results was used as the initial structure of the 200-ns molecular dynamics simulations and the preferential binding mode of ORO to α-HL was determined. The simulations show that ORO is a ligand that can bind to α-HL via hydrogen bonding and van der Waal interaction. Over the time course of the simulation, ORO localizes to the “Loop” region of α-HL, which is reported to participate in crucial protomer-protomer interactions during α-HL self-assembly and is therefore important in heptamer formation and cell lysis [6], [13]. The predicted binding mode of ORO with α-HL is illustrated in Figure 3A and the electrostatic potentials of the residues around the binding site are mapped, as shown in Figure 3B, using APBS software [14]. In detail, the binding model of ORO to the Loop of α-HL (Figure 3B) revealed that the methyl group of the 4H-chromen-4-one moiety of ORO formed a hydrogen bond with the side chain of the Lys46 amino acid in α-HL. As shown in Figure 4, the number of hydrogen bonds fluctuates mostly between 1 and 2 throughout the simulation time, which indicates that ORO and α-HL are always interacting via a hydrogen bond.

Moreover, the neutral side chains of the Thr11, Thr12, and Ile14 residues of α-HL form Van der Waals interactions with ORO, as shown in Figure 3B. Thr11 and Thr12 anchor the benzene ring of ORO, and Ile14 and Gly15 play an important role in stabilizing the 4H-chromen-4-one moiety of ORO. In addition, the methoxy of the 4H-chromen-4-one moiety forms strong interactions with Ser16 and Lys46, which will be confirmed by energy decomposition analysis.

**Figure 5:**
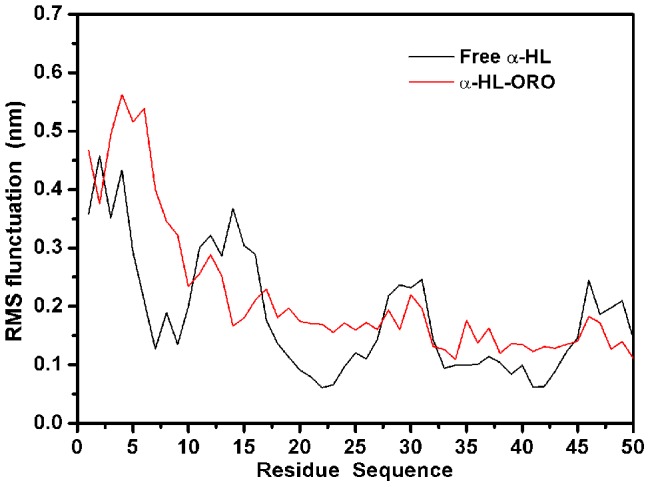
RMS fluctuations of the residues around the binding sites of the α-HL –ORO complex and free α-HL. RMS fluctuations are defined as RMS deviations of the structure at a given time from the average structure found using the MD simulation. Only mobile atoms of the simulation were considered for the RMS fluctuations.

The root mean square fluctuation (RMSF) of the residues surrounding the ORO binding site of α-HL (residues 1–50) in the α-HL-ORO complex and in free α-HL were calculated to illustrate the flexibility of these residues. The RMSF of these residues are shown in Figure 5 and clearly depict the difference in the flexibility of the binding site of α-HL due to the presence or absence of ORO. All of the residues in the α-HL binding site that is bound with ORO show a smaller degree of flexibility, with RMSF values less than 0.3 nm, when compared with the RMSF values calculated for the free α-HL, which indicates that these residues become more rigid after binding to ORO. These results indicate that the stabilization of the α-HL binding cavity in this complex is mostly due to residues Thr11, Thr12, Ile14, Gly15 and Lys46, as shown in Figure 3B.

### The binding site in the α-HL-ORO complex

**Figure 6:**
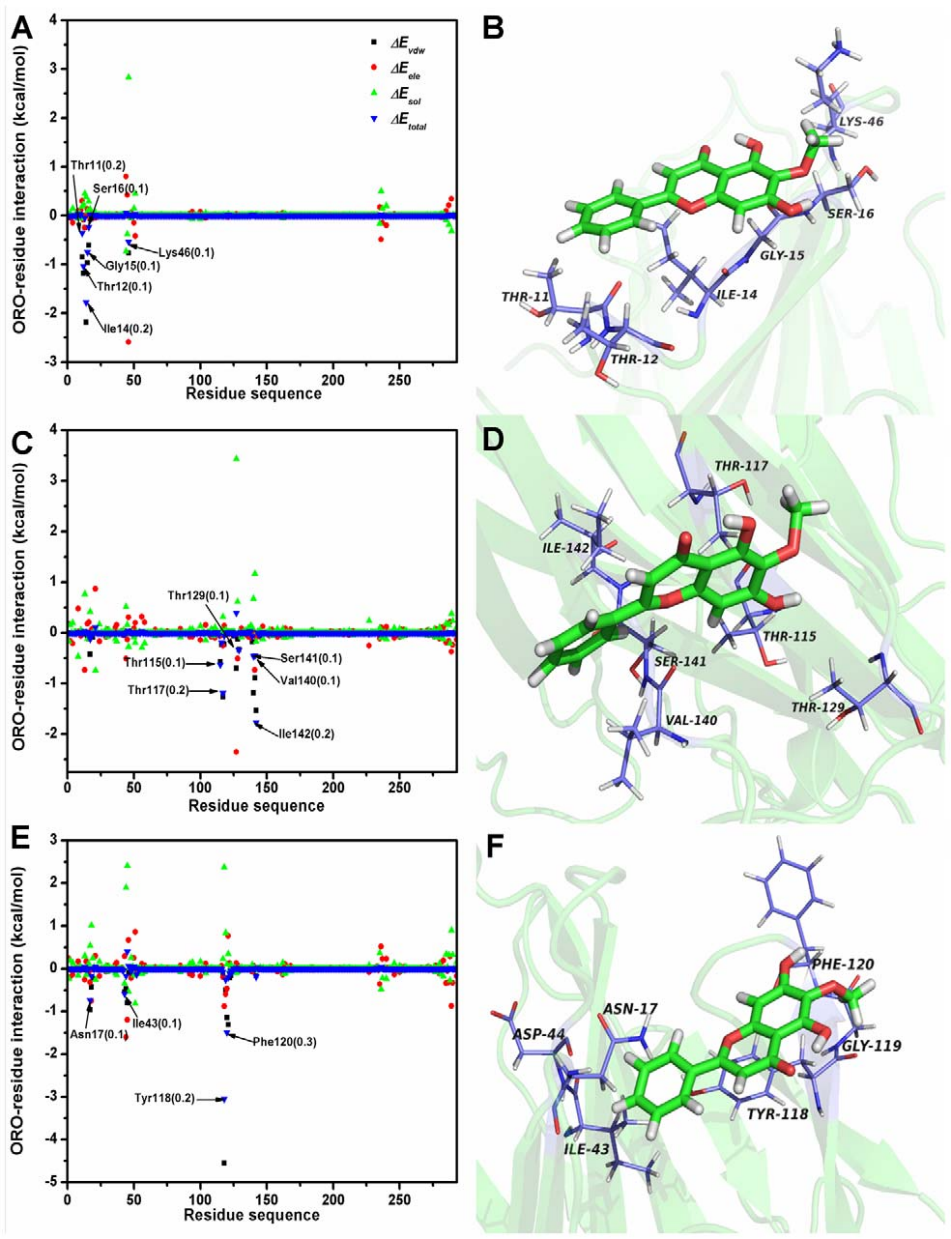
Energy contribution of van der Waals, electrostatic and solvation terms for the whole protein. (A), (B) Decomposition of the binding energy for each residue and predicted binding mode of ORO with the wild type protein. (C), (D) Decomposition of the binding energy for each residue and predicted binding mode of ORO with the T12A mutation. (E), (F) Decomposition of the binding energy for each residue and predicted binding mode of ORO with the I14A mutation. The error bars of *ΔE_total_* of the key residues have been given in the brackets.

The MD results provide an approximate binding mode of the protein-ligand interaction of the α-HL-ORO complex. However, the contribution of the residues surrounding the binding site of α-HL is not clear. Therefore, the electrostatic, Van der Waals, solvation and total contribution of the residues to the binding free energy were calculated using the Molecular Mechanics Generalized Born Surface Area (MM-GBSA) method [15], [16]. The calculation was performed over the 200 MD snapshots obtained from the last 50-ns simulation. The summations of the interaction free energies for each residue were separated into Van der Waals (*ΔE_vdw_*), electrostatic (*ΔE_ele_*), solvation (*ΔE_sol_*), and total contribution (*ΔE_total_*). The energy contributions from the all residues of α-HL are summarized in Figure 6A.

As shown, Lys46 has an appreciable electrostatic (*ΔE_ele_*) contribution, with a value less than −2.3 kcal/mol (Figure 6A and 6B). Because Lys46 is close to the methoxy of ORO and an electrostatic interaction exists, strong H-bonds are formed between α-HL and ORO. In addition, residues Thr12 (with a *ΔE_vdw_* of −1.2 kcal/mol) and Ile14 (with a *ΔE_vdw_* of ≤−2.1) exhibit strong Van der Waals interactions with the ligand because of the close proximity between these two residues and the 4H-chromen-4-one moiety of ORO. The majority of the decomposed energy interaction, with the exception of the energy associated with Lys46, originates from Van der Waals interactions. The electrostatic contribution from these key residues does not appear to have a significant influence on the formation of the α-HL-ORO complex.

The total binding free energy of the α-HL-ORO complex, *ΔG_bind_*, and its detailed energy contributions, which were calculated using the MM-GBSA approach, are summarized in Table 1. The *ΔG_bind_* can be divided into polar (*ΔG_ele,sol_*+*ΔE_ele_*) and nonpolar (*ΔG_nonpolar_*+*ΔE_vdw_*) energies. As shown, the primary contributor to the free energy of the binding of ORO to α-HL is *ΔG_nonpolar_*+*ΔE_vdw_*, with a value of −11.2 kcal/mol, whereas *ΔG_ele,sol_*+*ΔE_ele_* have a minor contribution of −6.4 kcal/mol. This difference arises from the intermolecular Van der Waals energy, which is mainly achieved from the ORO-binding α-HL residues. After summation of the solute entropy term (5.1 kcal/mol), an estimated *ΔG_bind_* of −12.5 kcal/mol was found, which suggests that ORO can strongly bind to and interact with the binding site of α-HL.

**Table 1:** Calculated energy components, binding free energy (kcal/mol) of ORO binding to active site of α-HL.

Energy components (kcal/mol)	WT	T12A	I14A
*ΔE_ele_*	−2.3±0.7	−3.1±0.8	−2.9±0.6
*ΔE_vdw_*	−25.0±1.2	−13.8±1.6	−16.8±1.4
*ΔE_MM_*	−27.3±3.1	−16.9±3.3	−24.1±2.2
*ΔG_ele,sol_*	−4.1±0.2	−0.8±0.2	−2.3±0.8
*ΔG_nonpolar,sol_*	13.8±2.7	9.2±2.8	11.7±2.3
*ΔG_sol_*	16.9±2.7	8.1±2.7	9.6±2.2
*ΔG_ele,sol_*+*ΔE_ele_*	−6.4±1.3	−3.9±1.3	−5.2±0.9
*ΔG_nonpolar_*+*ΔE_vdw_*	−11.2±0.6	−4.6±0.7	−5.1±1.7
*ΔG_total_*	−17.6±1.6	−8.5±2.8	−10.3±1.4
*−TΔS*	5.1±1.3	4.7±1.1	5.1±2.4
*ΔG_bind_*	−12.5±1.9	−3.8±1.7	−5.2±1.5

The same procedure was followed with two α-HL mutants, T12A-HL and I14A-HL, to verify the accuracy of the binding site in the α-HL-ORO complex. A complex of each mutant with ORO was used as the preliminary structure for the MD simulations and the MD trajectories were analyzed using the MM-GBSA method. In addition, the T12A-HL and I14A-HL mutants were expressed and purified; the binding free energy and the number of binding sites between ORO and the two mutants were then determined using the fluorescence spectroscopy quenching method [17], [18].

As illustrated in Figure 6D and 6F, ORO differentially binds to the two mutants and the WT-HL, an observation that was confirmed by pair interaction decomposition analysis of the free energy, as shown in Figure 6C and 6E. The major contributions to the free energy of the complexes of ORO with the α-HL mutants were residues Thr115, Tyr118, Pro103, Phe120 and Ile142. Furthermore, as shown in Table 1, the MM-GBSA calculation predicted that T12A-HL and I14A-HL bound more weakly to ORO than WT-HL, with estimated *ΔG_bind_* values of −3.8 kcal/mol and −5.2 kcal/mol, respectively. Consequently, the calculations for T12A-HL and I14A-HL show that these mutants exhibited a decrease in the binding energy of approximately 7 to 8 kcal/mol compared to WT-HL. The experimental measurement of the binding free energy, shows that the interaction between ORO and WT-HL is highest, which means that WT-HL has the strongest ability to bind to ORO; the mutants exhibits the weaker ability, as shown in Table 2. Importantly, because the calculated binding free energies are in good agreement with the experimental data shown in Table 2, we believe that the MD simulations generated a reliable model of the α-HL-ORO complex.

**Table 2:** Values of the binding free energy and the number of binding sites (n) of α-HL-ORO systems based on the fluorescence-quenching method.

	WT	T12A	I14A
*ΔG_bind_* (kcal/mol)	8.4±0.2	6.3±0.1	6.9±0.1
n	1.0719	0.9945	0.9751

### The conformation change of the “Loop” in the complex

**Figure 7:**
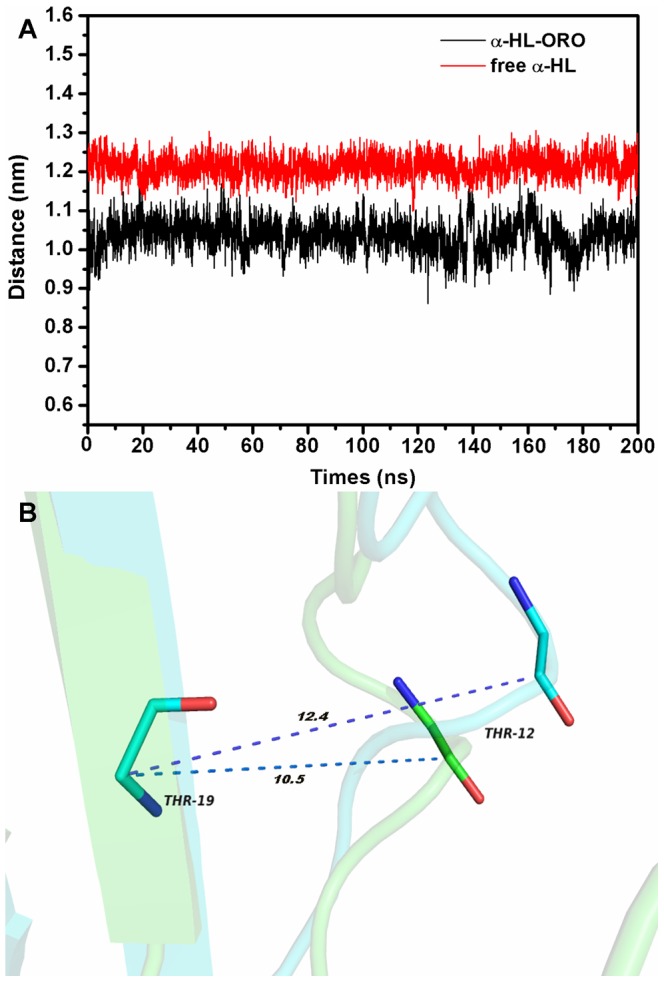
Comparison of the distance between the C_α_ atoms of Thr12 and Thr19 in different systems. (A) The distance between the C_α_ atoms of Thr12 and Thr19 as a function of time. (B) The distances between the C_α_ atoms of Thr12 and Thr19 is shown in the superimposition of the structures of free α-HL (blue) and the α-HL-ORO complex (green). The average distance in the complex and in the free protein is 1.05 nm and 1.24 nm, respectively.

**Figure 8:**
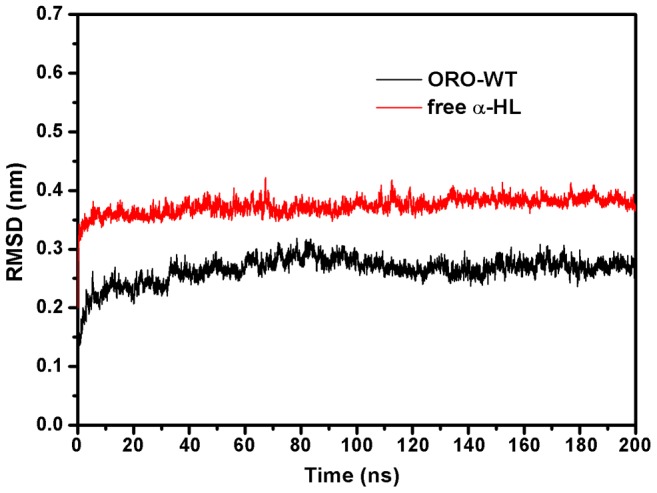
RMSD of C_α_ atoms of “Loop” from their initial coordinates as a function of time. MD simulations of the α-HL-ORO complex displayed significantly decreased dynamics in the “Loop”. The two systems were found to reach equilibrium after 10 nanoseconds and displayed the following RMSD trends: RMSD*_free_*>RMSD*_wt_*.

**Figure 9:**
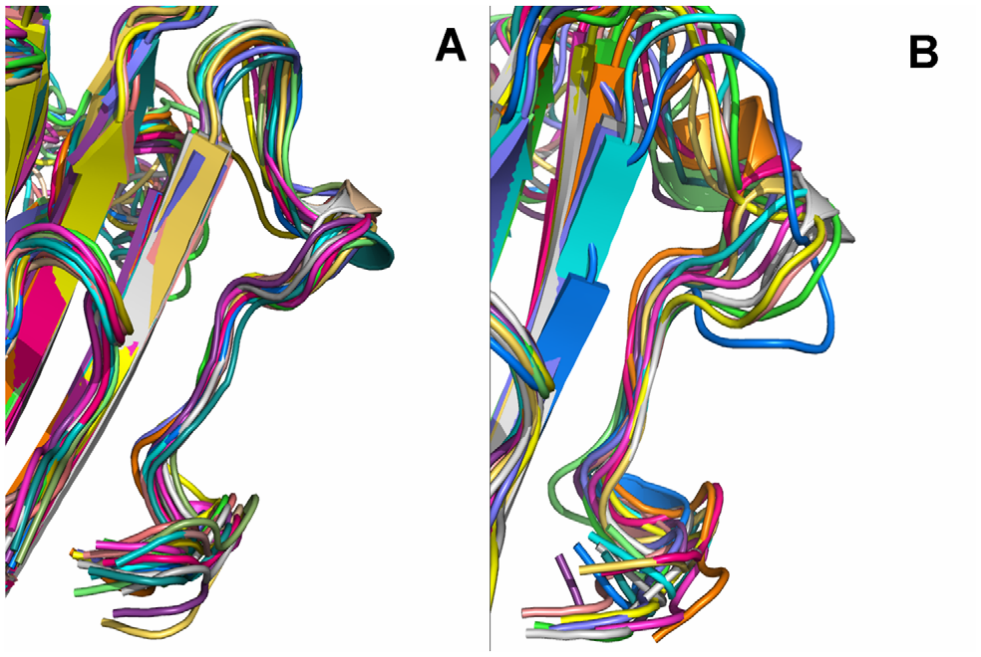
Significant differences were displayed in the dynamics of the critical “Loop” region during the last 20-ns simulations of the wild type-ORO and the free wild-type α-HL. Ribbon diagrams of the conformations of the loop extracted from MD simulations are presented. The “Loop” is located below each image. 30 snapshots from the wild-type's MD simulation are displayed in (A); the free protein are displayed in (B). The snapshots were superimposed using the backbone atoms by Pymol software.

ORO inhibits the hemolytic activity of α-HL and binds to the “Loop” cavity of α-HL, which has been shown to be critical to its hemolytic activity [6], [19]. Consequently, the conformations of the “Loop” region in the α-HL-ORO complex and in free α-HL were investigated using a MD simulated trajectory. As shown in Figure 7A, the distance between the C_α_ of Thr12 and the C_α_ of Thr19 in the complex ranged from 0.9 to 1.15 nm over the time course of the simulation, with an average distance of 1.05 nm (Figure 7A and 7B). In the absence of ORO, the distance between these points ranges from 1.15 to 1.3 nm, with an average distance of 1.24 nm. However, the distance between the C_α_ of Thr12 and the C_α_ of Thr19 is 1.81 nm in the crystal structure of the α-HL heptamer, which is available in the Protein Data Bank under the accession number 7AHL. Dynamic fluctuations in the distance between the C_α_ of Thr12 and the C_α_ of Thr19 likely indicate that the conformation of the “Loop” region is restrained when ORO binds to these two residues. Through comparing the structure of the loop in α-HL-ORO complex with the crystal structure of α-HL monomeric observed in the X-ray structure of the oligomer (PDB code: 7AHL), it is indicated that ORO blocks the required conformational transition of the loop by binding to the loop, which is the mechanism of decreasing the lytic activity of α-HL. The RMSD values also reflect the variation in the conformation change of the “Loop” region during the simulation time. As shown in Figure 8, the RMSD values of the “Loop” region in free α-HL are ∼0.4 nm; these values are clearly higher than that of the α-HL-ORO complex, which is in the range of 0.323 nm. In addition, as shown by the RMSD trends displayed in Figure 8, the RMSD data agreed with other types of measurements, reinforcing that the WT-HL and ORO complex displays very little variation in the conformation of the “Loop” during the MD simulation. Furthermore, significant differences were observed in the dynamics of the critical “Loop” region during the MD simulations of the wild type and free α-HL, which confirms the effect of inhibition of α-HL on cell lysis. As illustrated in Figure 9, our models predict a marked conformational transition in the “Loop” region for the wild type complex to the free α-HL.

**Figure 10:**
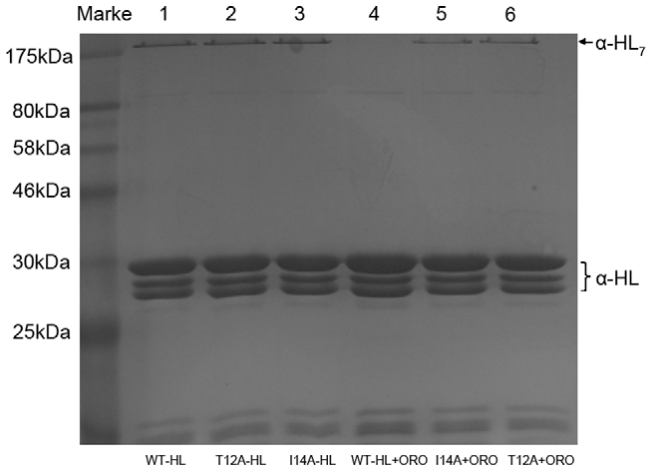
ORO prevents the deoxycholate-induced oligomerization of α-HL. α-HL was treated with 5 mM deoxycholate in the presence or absence of ORO. Following sodium dodecyl sulfate (SDS)-polyacrylamide gel electrophoresis (PAGE) analysis, the proteins were detected by silver staining. Lane 1, WT-HL; lane 2, T12A-HL; lane 3, I14A-HL; lane 4, WT-HL plus 8 µg/ml of ORO; lane 5, I14A-HL plus 8 µg/ml of ORO; lane 6, T12A-HL plus 8 µg/ml of ORO.

A comparative analysis of these MD simulations suggests that the inhibitory activity of ORO is highest for the wild type α-HL, followed by the T12A mutant and then I14A. This conclusion, which is also supported by the mechanism of hemolytic inhibition, is in good agreement with experimental results. Data from a deoxycholate-induced oligomerization assay shows that the site-directed mutagenesis of T12A and I14A has no influence on the assembly of the SDS-stable oligomer, α-HL_7_. However, the formation of α-HL_7_ was inhibited when treated with 8 µg/ml of ORO. This inhibitory effect was decreased with either of the two mutants, as shown in Figure 10, with I14A showing a higher inhibition than T12A.

These findings support one possible inhibition mechanism: the binding of ORO to the “Loop” region blocks the conformational change, which inhibits the self-assembly of the heptameric transmembrane pore, thereby decreasing the lytic activity of α-HL.

### ORO prevents α-HL-mediated A549 cell injury

**Figure 11:**
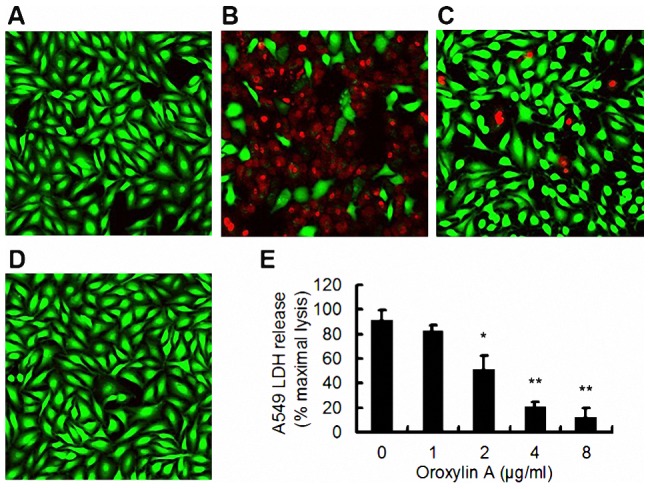
ORO protects A549 cells from α-HL mediated cell injury. Live (green)/dead (red) stained A549 cells were captured by confocal laser scanning microscopy after infection with *S. aureus* 8325-4 or DU 1090. Uninfected cells (A); cells co-cultured with *S. aureus* 8325-4 in the absence of ORO (B); cells co-cultured with *S. aureus* 8325-4 in the presence of 8 µg/ml of ORO (C); cells infected with DU1090 (D); (E) LDH release by A549 cells when co-cultured with *S. aureus* 8325-4 and the indicated concentrations of ORO. All data represent the mean and standard error of three independent experiments. * indicates P<0.05 and ** indicates P<0.01 compared to the drug-free culture.

Human alveolar epithelial (A549) cells have previously been employed to investigate the influence of *S. aureus* on lung cell injury and α-HL has been found to be the major factor associated with their injury and death [7]. Because our previous results show that ORO blocks the self-assembly of α-HL, we speculated that ORO may protect A549 cells from *S. aureus*-mediated death. Consequently, A549 cells were stained with a live/dead (green/red) reagent following co-culture with *S. aureus* 8325-4. The uninfected cells displayed the green fluorophore (Figure 11A), indicating their live status. A549 cells were significantly affected by their co-culture with *S. aureus*, as reflected by the increase in the amount of red fluorophore observed (Figure 11B). However, the addition of 8 µg/ml of ORO resulted in a significantly lower number of dead cells (Figure 11C). Consistent with a previous study, treatment of A549 cells with the *S. aureus* strain DU1090, which cannot produce α-HL, does not result in their death (Figure 11D) [11]. Furthermore, a lactate dehydrogenase (LDH) release assay was employed to quantitatively assess the influence of ORO on the protection of A549 cell injury and, as shown in Figure 11E, the addition of 1 to 8 µg/ml of ORO affords a dose-dependent protection. The 50% inhibitory concentration (IC_50_), which was calculated using OriginPro 8.0 (OriginLab, USA), was 3.09 µg/ml. These results highlight the potential therapeutic effect of ORO, which merits further investigation.

## Discussion

Historically, vancomycin and linezolid have been the recommended empirical and definitive therapies for the treatment of methicillin-resistant *S. aureus* pneumonia. However, the emergence of multi-drug-resistant *S. aureus*, such as vancomycin-resistant *S. aureus*, makes *S. aureus* infection difficult to treat and increases its mortality rate [20]. Due to our increasing understanding of bacterial pathogenesis and intercellular cell signaling, several potential strategies have been developed for drug discovery, of which the anti-virulence strategy has the interest of most researchers [21]. *S. aureus* can secrete numerous surface proteins and exotoxins, which are involved in the process of pathopoiesis [22]. One of the most important is α-HL, which often leads to tissue damage. Our previous research has also shown that several natural compounds can protect mice against *S. aureus* pneumonia by decreasing the production of α-HL [11]. Based on the results of all these studies, we theorized that α-HL can be used as a target for the development of new drugs against *S. aureus*.

The increasing interest in drug design based on the identification of novel virulence targets has created a demand for the structural characterization of protein-ligand complexes. X-ray crystallography is a traditional tool used to investigate the interaction of ligands and proteins in a complex, and many studies using this technique have been reported. In 1996, Song et al. discovered the crystal structure of the α-HL heptamer; however, to date, the monomeric structure of α-HL remains unknown, suggesting that crystals of the α-HL monomer may be very difficult to obtain. Thus, it seems impractical to explore the structure of the ORO-α-HL monomer complex by crystallography. In the literature, computational chemistry combined with experimental confirmation has proven an effective and reliable method for exploring the interactions between ligands and proteins. Accordingly, in this study, we used a complementary approach that includes molecular dynamics simulations (MD simulations), site-specific mutagenesis, and a fluorescence-quenching method to further explore ligand-protein binding sites. Specifically, we attempted to identify the mechanism by which ORO inhibits the biological activity of α-HL.

Moreover, to explore the formation of the interaction between a protein and a ligand at the atomic level, we used the MM-GBSA method to determine the associated free energy profiles. It has been known for years, and was restated recently [23], that free energy calculations from MD simulations can prove to be a powerful tool for exploring the process of ligand-protein binding when used in combination with mutagenesis experiments [24]–[27]. In addition, MM-GBSA calculations were performed on a series of derivatives of TIBO (a substituted tetrahydroimidazole benzodiazepine thione) to explore their potential as inhibitors of HIV-1 reverse transcriptase [28]. In the same study, the binding mode of a known drug was predicted with excellent agreement to the X-ray structure, which was discovered afterward. Other examples of studies with MD simulations include work by Biswa Ranjan Meher et al. [29], who examined the binding of the inhibitor darunavir to wild-type and mutant proteins using all-atom MD simulations and MM-GBSA calculations, and work by Lstyastono et al. [30], whose study focused on the elucidation of molecular determinants of G protein-coupled receptor-ligand binding modes by combining MD simulations and site-directed mutagenesis studies. There are a number of other such examples of studies that employ MD simulations [31]–[36]. In this study, we discovered that Van der Waals interactions play an important role in the stabilization of the binding site of α-HL-ORO. The key residues, Thr11, Thr12, Ile14, Gly15 and Lys46, in the complex were also identified, using residue decomposition analysis and mutagenesis assays. The conformational transition of the critical “Loop” region from the monomeric α-HL to the oligomer was blocked by the binding of ORO, which resulted in the inhibition of the hemolytic activity of α-HL. These findings indicate that ORO hinders the lytic activity of α-HL through a novel mechanism. This was confirmed by inducing the formation of the α-HL heptamer by Deoxycholate, the results of which indicate that addition of ORO inhibits the formation of the α-HL heptamer.

In summary, we found that oroxylin A (ORO), a natural compound without anti-*S. aureus* activity, can inhibit the hemolytic activity of α-HL. Based on the results of MD simulation, we confirmed that ORO could inhibit the hemolytic activity of α-HL by a new mechanism which is completely different compared with baicalin (BAI). Through the analysis of the binding free energy of the complex formation using MM-GBSA method, the results show that the residues Thr11, Thr12, Ile14, Gly15 and Lys46, which surround the binding site of the α-HL-ORO complex, are key ORO-binding residues. Due to the binding of ORO, the conformation transition of the critical “Loop” region from the monomeric α-HL to the oligomer was blocked, which resulted in inhibition of the hemolytic activity of the protein. This novel mechanism was confirmed by experimental data using a deoxycholate-induced oligomerization assay. The whole results mentioned above indicate that the “triangle region” of α-HL is not the only active site of inhibitor and “Loop” region of α-HL also plays an important role in the inhibition of hemolytic activity of α-HL, which could facilitate the design of new and more effective antibacterial agents.

## Materials and Methods

### Micro-organism, reagents and animals

*S. aureus* 8325-4, a high-level α-HL-producing strain, and its cognate α-HL-deficient mutant, DU 1090, were used in this study. The *S. aureus* strains 8325-4 and DU 1090 were cultured in TSB to an optical density of 0.5 at 600 nm. Then, either the cultures were centrifuged and resuspended in DMEM medium for the live/dead and cytotoxicity assays. ORO (purity>98.5%) (Figure 2A) was obtained from Sigma-Aldrich (St. Louis, MO, USA), and the stock solutions were prepared in dimethyl sulfoxide (DMSO) (Sigma-Aldrich). For the in vivo studies, ORO was dissolved in sterile PBS. The minimum inhibitory concentrations (MICs) of ORO for *S. aureus* were evaluated using the broth microdilution method according to the Clinical and Laboratory Standards Institute (CLSI) guidelines.

### Hemolysis assay

Hemolytic activity was measured as described elsewhere using rabbit red blood cells [7]. In brief, 100 µl of purified α-HL was pre-incubated in 96-well microtiter plates in the presence of either gradient concentrations of ORO or PBS control at 37°C for 10 min. Defibrinated rabbit red blood cells (100 µl; 5×10^6^ cells per milliliter) in PBS were then added to the wells and the mixtures were incubated at 37°C for 20 min using 1% Triton X-100 as a positive control. After centrifugation, the supernatants were removed and their absorption at 543 nm was measured. The percent hemolysis was calculated using the supernatant reading from an equivalent number of cells that had been lysed in 1% Triton X-100.

### Immunoblot analysis

Western blot analysis was performed as previously described [37]. Briefly, *S. aureus* 8325-4 and DU 1090 were cultured at 37°C in TSB and different concentrations of ORO to an optical density at 600 nm of 2.5. The cultures were collected by centrifugation and the supernatants were used in sodium dodecyl sulfate (SDS)-polyacrylamide (12%) gel electrophoresis. The proteins were then transferred onto polyvinylidene fluoride membranes (Roche, Basel, Swiss) using a semi-dry transfer cell (Bio-Rad, Munich, Germany). After blocking the membrane for 2 h with 5% Bovine Serum Albumin (BSA) (Amresco, USA) at room temperature, an anti-hemolysin primary polyclonal antibody (Sigma-Aldrich) was added at a 1 ∶ 5000 dilution. The membrane was then incubated overnight at 4°C and then for 2 h with a HRP-conjugated secondary goat anti-rabbit antiserum (Sigma-Aldrich) that was diluted to 1 ∶ 4000. The blots were developed using Amersham ECL western blotting detection reagents (GE Healthcare, UK).

### Homology modeling of α-HL

For rational drug discovery, modeling and informatics play an indispensable role in the identification of lead compounds and their most plausible mechanisms of action against particular biological targets [38]. Therefore, we have performed a homology-modeling study on α-HL. To date, the structure of monomeric α-HL is unavailable and only the crystal structure of the α-HL heptamer has been reported [6].

The model of the monomeric α-HL in solution was proposed based on homology modeling, as previously reported [12]. MODELLER [3], version 9.9, was used to generate structural models of α-HL based on the template structures of LukF (PDB codes 1LKF_A), LukF-PV (PDB code 1PVL_A), Gamma-hemolysin component A (PDB code 2QK7_A) and LukS-PV (PDB code 1T5R_A). As a whole, the sequence identities between the templates Lukf-PV, LukF, Gamma-hemolysin component A, LukS-PV and the query monomeric α-HL are 30%, 31%, 26%, and 22%. The most important difference between template and query is located in “loop” region (residues 1–50), which is the critical region for the assembled hemolysin pore. The program optimizes the structure of the homology models by minimizing a global probability density function that integrates the stereochemical parameters and homology-derived restraints [39]. The best model was selected based on its DOPE score, and it was subjected to further 200 ns molecular dynamics using Gromacs 4.5.1 software package [40].

The geometry of ORO was optimized at the B3LYP/6-31G* level using the Gaussian 03 program [41].

### Molecular docking calculation

The initial structure of α-HL was obtained from the homology modeling. To obtain the starting structure of the drug/α-HL complex for molecular dynamics (MD) simulation, a standard docking procedure for a rigid protein and a flexible ligand was performed with AutoDock 4 [42], [43]. The Lamarckian genetic algorithm (LGA) was applied in the docking calculations. All of the torsional bonds of the drug were free to rotate while α-HL was held rigid. Then, the polar hydrogen atoms were added for α-HL using the AutoDock tools, and Kollman united atom partial charges [44] were assigned. A total of 150 independent runs were carried out with a maximum of energy evaluations to 25,000,000 and a population size to 300. A grid box (50×40×49) with spacing of 0.1 nm was created and centered on the mass center of the ligand. Energy grid maps for all possible ligand atom types were generated using Autogrid 4 before performing the docking.

The clusters were ranked according to the lowest energy representative in each cluster. Then, the ligand docking poses suggesting preferential binding to the loop region are three: Pose 1, Pose 2, and Pose 3. Pose 1 has the lowest energy conformation (−6.5 kcal/mol) and the most populated cluster (28) compared with Pose 2 (−5.6 kcal/mol, 15) and Pose 3(−4.9 kcal/mol, 7), and then Pose 1 was chosen for further study. The lowest energy conformation in the most populated cluster was chosen for further study [45].

### Molecular dynamics simulations

All of the simulations and the analysis of the trajectories were performed with Gromacs 4.5.1 software package using the Amber ff99sb force field and the TIP3P water model [40], [46]. The α-HL-ORO system was first energy relaxed with 2000 steps of steepest-descent energy minimization followed by another 2000 steps of conjugate-gradient energy minimization. The system was then equilibrated by a 500 ps of MD run with position restraints on the protein and ligand to allow for relaxation of the solvent molecules. The first equilibration run was followed by a 200 ns MD run without position restraints on the solute. The first 20 ns of the trajectory were not used in the subsequent analysis to minimize convergence artifacts. The equilibration of the trajectory was checked by monitoring the equilibration of quantities, such as the root-mean-square deviation (RMSD) with respect to the initial structure, the internal protein energy, and fluctuations calculated for different time intervals. The electrostatic term was described with the particle mesh Ewald algorithm. The LINCS [47] algorithm was used to constrain all bond lengths. For the water molecules, the SETTLE algorithm [48] was used. A dielectric permittivity, *ε* = 1, and a time step of 2 fs were used. All atoms were given an initial velocity obtained from a Maxwellian distribution at the desired initial temperature of 300 K. The density of the system was adjusted during the first equilibration runs at *NPT* condition by weak coupling to a bath of constant pressure (*P*_0_ = 1 bar, coupling time *τ_P_* = 0.5 ps) [49]. In all simulations, the temperature was maintained close to the intended values by weak coupling to an external temperature bath with a coupling constant of 0.1 ps. The proteins and the rest of the system were coupled separately to the temperature bath. The structural cluster analysis was carried out using the method described by Daura and co-workers with a cutoff of 0.25 nm [48].

The ORO parameters were estimated with the antechamber programs [49] and AM1-BCC partial atomic charges from the Amber suite of programs [50].

Analysis of the trajectories was performed using PyMOL analysis tools and Gromacs analysis tools.

### Calculation of binding free energy

In this work, the binding free energies are calculated using MM-GBSA approach supplied with Amber 10 package. We choose a total number of 200 snapshots evenly from the last 50 ns on the MD trajectory with an interval of 10 ps. The MM-GBSA method can be conceptually summarized as:
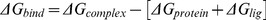
(1)

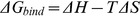
(2)where *ΔH* of the system is composed of the enthalpy changes in the gas phase upon complex formation (*ΔE_MM_*) and the solvated free energy contribution (*ΔG_sol_*), while *−TΔS* refers to the entropy contribution to the binding. Eq. (2) can be then approximated as shown in Eq. (3):
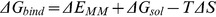
(3)where *ΔE_MM_* is the summation of the van der Waals (*ΔE_vdw_*) and the electrostatic (*ΔE_ele_*) interaction energies.
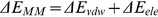
(4)In addition, *ΔG_sol_*, which denotes the solvation free energy, can be computed as the summation of an electrostatic component (*ΔG_ele,sol_*) and a nonpolar component (*ΔG_nonpolar,sol_*), as shown in Eq. (5):
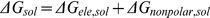
(5)


### Ligand-residue interaction decomposition

The interactions between ORO and the all residues of α-HL are analyzed using the MM-GBSA decomposition process applied in the MM-GBSA module in Amber **10.** The binding interaction of each ligand-residue pair includes three terms: the Van der Waals contribution (*ΔE_vdw_*), the electrostatic contribution (*ΔE_ele_*), and the solvation contribution (*ΔE_sol_*). All energy components are calculated using the same snapshots as the free energy calculation.

### Mutagenesis of the α-HL protein

#### (i) Construction of plasmids encoding wild-type (WT)-HL, T12A-HL and I14A-HL

*S. aureus hla* gene, which encodes a mature protein of 293 amino acids, was amplified using the genomic DNA of *S. aureus* strain 8325-4 as a template and the following primers:

5′-CGCGGATCCGCAGATTCTGATATTAATATTAAAAC-3′ (forward) and 5-′CCGCTCGAGTTAATTTGTCATTTCTTCTTTTTC-3′ (reverse). Two restriction endonuclease sites BamHI and XhoI were attached to the 5′ ends of the upstream and downstream primer to facilitate subsequent cloning, respectively. Following digestion, the digested PCR product was cloned into the prokaryotic expression vector pGEX-6P-1 (Merck Biosciences, Germany) with BamHI and XhoI restriction enzyme sites, resulting in the recombinant plasmid pGEX-6P-1-hla, encoding WT-HL.

Plasmids encoding T12A-HL or I14A-HL were constructed using the QuikChange site-directed mutagenesis kit (Stratagene, CA, USA) from the template plasmid pGEX-6P-1-*hla*. The mutagenic primers for T12A-HL were 5′-GATATTAATATTAAAACCGGTACTGCAGATATTGGAAGCAATACTAC-3′(forward) and 5′-GTAGTATTGCTTCCAATATCTGCAGTACCGGTTTTAATATTAATATC-3′ (reverse). The mutagenic primers for I14A-HL were 5′-CCGGTACTACAGATGCTGGAAGCAATACTAC-3′ (forward) and 5′-GTAGTATTGCTTCCAGCATCTGTAGTACCGG-3′ (reverse). The codons that were modified are underlined in each primer sequence.

#### (ii) Expression and purification of WT-HL, T12A-HL and I14A-HL

The recombinant plasmid harboring the coding sequences for WT-HL, T12A-HL and I14A-HL was transformed into *Escherichia coli* strain BL21 (DE3). Protein expression was induced by addition of 0.2 mM IPTG when the OD600 of the culture reached approximately 0.6–0.8 in LB medium. The cultures were allowed to grow for further 20 h at 16°C. Cells were harvested by centrifugation at 5,000×g for 10 min at 4°C, resuspended in lysis buffer (1×PBS, 1 mM DTT and 1 mM PMSF) and lysed by sonication. Cell lysates were centrifuged at 15, 000 rpm for 40 min at 4°C. The supernatant of each lysate was loaded onto a self-packed GST-affinity column (2 mL glutathione-Sepharose 4B) (GE Amersham). The contaminant proteins were washed away with wash buffer (lysis buffer with 200 mM NaCl). Cleavage of GST tag was achieved by PreScission protease (GE Amersham) overnight at 4°C. The proteins were eluted with lysis buffer with an additional five amino acid tag (GPLGS) at the N-terminus. The eluant was desalted using a Sephadex G-25 column (GE Healthcare) with buffer A (25 mM MES, pH 6.0). The desalted proteins were purified using a Resource S column (GE Healthcare) with buffer A and buffer B (25 mM MES, 1 M NaCl, pH 6.0). For further purification, a Superdex 75 16/60 column (GE Healthcare) was used with buffer A. The purified proteins were analyzed by SDS-PAGE and MALDI-TOF MS. The fractions containing pure target protein were pooled and stored at −80°C for subsequent applications.

### Binding affinity determination of ORO with WT-HL, T12A-HL and I14A-HL

The binding constants (*K_A_* of ORO to the binding site on WT-HL, T12A-HL and I14A-HL were measured using the fluorescence-quenching method, and the binding constants were converted to the binding energy by Eq. *ΔG_bind_* = RTln*K_A_*. Fluorescence spectrofluorimetry measurements were carried out using a Horiba Jobin-Yvon Fluorolog 3–221 spectrofluorometer (Horiba Jobin-Yvon, Edison, NJ). The measurements were acquired using a 280-nm excitation wavelength with a 5-nm band-pass and a 345-nm emission wavelength with a 10-nm band-pass. Details of the measurements were described previously [51]–[53].

### Oligomerization assay

Oligomerization assay was performed as described previously [54], 200 ng WT-HL, T12A-HL or I14A-HL monomers was mixed with 5 mM deoxycholate separately, following the addition of ORO, the mixtures were incubated at 22°C for 20 min. Then 5× loading buffer without β-mercaptoethanol was added to the mixtures and incubated at 50°C for 10 min. 25 µl of each reaction mixture was loaded onto 12% sodium dodecyl sulfate (SDS)-polyacrylamide gel electrophoresis (PAGE) gels for electrophoresis. Gels were stained using the silver PlusOne staining kit (GE Healthcare) according to the manufacturer's instruction.

### Live/dead and cytotoxicity assays

Human lung epithelial cells (A549) were obtained from the American Tissue Culture Collection (ATCC CCL 185) and cultured in Dulbecco's modified Eagle's medium (DMEM) (Invitrogen, CA, USA) supplemented 10% fetal bovine serum (Invitrogen). Cells were seeded in 96-well dishes at a density of approximately 2×10^5^ cells each well. As described previously [55], A549 cells were incubated with 100 µl of staphylococcal suspension with the addition of different concentrations of ORO or positive control PBS for 6 h at 37°C, DU1090 suspension was used as negative control. Cell viability was determined either using live/dead (green/red) reagent (Invitrogen) or by measuring lactate dehydrogenase (LDH) release using a Cytotoxicity Detection kit (LDH) (Roche) according to the manufacturer's directions. Microscopic images of stained cells were obtained using a confocal laser scanning microscope (Nikon, Japan). LDH activity was measured on a microplate reader (TECAN, Austria).

### Statistical analysis

The significance of hemolysis, LDH release assay results were determined using the two-tailed Student's t test. Differences were considered statistically significant when P<0.05.

### Accession numbers

*S. aureus hla* gene: NC_007795.1

## References

[1] Lowy FD (1998) *Staphylococcus aureus* infections. N Engl J Med 339: 520–532. 9709046 10.1056/NEJM199808203390806

[2] Sutra L, Poutrel B (1994) Virulence factors involved in the pathogenesis of bovine intramammary infections due to *Staphylococcus aureus* . J Med Microbiol 40: 79–89. 8107066 10.1099/00222615-40-2-79

[3] Aksimentiev A, Schulten K (2005) Imaging alpha-hemolysin with molecular dynamics: ionic conductance, osmotic permeability, and the electrostatic potential map. Biophys J 88: 3745–3761. 15764651 10.1529/biophysj.104.058727 PMC1305609

[4] Bhakdi S, Tranum-Jensen J (1991) Alpha-toxin of *Staphylococcus aureus.* . Microbiol Rev 55: 733–751. 1779933 10.1128/mr.55.4.733-751.1991 PMC372845

[5] Bartlett AH, Foster TJ, Hayashida A, Park PW (2008) Alpha-toxin facilitates the generation of CXC chemokine gradients and stimulates neutrophil homing in *Staphylococcus aureus* pneumonia. J Infect Dis 198: 1529–1535. 18823272 10.1086/592758 PMC12822823

[6] Song L, Hobaugh MR, Shustak C, Cheley S, Bayley H, et al (1996) Structure of staphylococcal alpha-hemolysin, a heptameric transmembrane pore. Science 274: 1859–1866. 8943190 10.1126/science.274.5294.1859

[7] Ragle BE, Karginov VA, Bubeck Wardenburg J (2010) Prevention and treatment of *Staphylococcus aureus* pneumonia with a beta-cyclodextrin derivative. Antimicrob Agents Chemother 54: 298–304. 19805564 10.1128/AAC.00973-09 PMC2798498

[8] Gu LQ, Bayley H (2000) Interaction of the noncovalent molecular adapter, beta-cyclodextrin, with the staphylococcal alpha-hemolysin pore. Biophys J 79: 1967–1975. 11023901 10.1016/S0006-3495(00)76445-9 PMC1301087

[9] Gu LQ, Braha O, Conlan S, Cheley S, Bayley H (1999) Stochastic sensing of organic analytes by a pore-forming protein containing a molecular adapter. Nature 398: 686–690. 10227291 10.1038/19491

[10] Qiu J, Niu X, Dong J, Wang D, Wang J, et al (2012) Baicalin protects mice from *Staphylococcus aureus* pneumonia via inhibiting the cytolytic activity of α-hemolysin. J Infect Dis 206: 292–301. 22551812 10.1093/infdis/jis336

[11] Qiu J, Luo M, Wang J, Dong J, Li H, et al (2011) Isoalantolactone protects against *Staphylococcus aureus* pneumonia. FEMS Microbiol Lett 324: 147–155. 22092816 10.1111/j.1574-6968.2011.02397.x

[12] Meesters C, Brack A, Hellmann N, Decker H (2009) Structural characterization of the alpha-hemolysin monomer from *Staphylococcus aureus* . Proteins 75: 118–126. 18798569 10.1002/prot.22227

[13] Walker B, Bayley H (1995) Key residues for membrane binding, oligomerization, and pore forming activity of staphylococcal alpha-hemolysin identified by cysteine scanning mutagenesis and targeted chemical modification. J Biol Chem 270: 23065–23071. 7559447 10.1074/jbc.270.39.23065

[14] Baker NA, Sept D, Joseph S, Holst MJ, McCammon JA (2001) Electrostatics of nanosystems: application to microtubules and the ribosome. Proc Natl Acad Sci USA 98: 10037–10041. 11517324 10.1073/pnas.181342398 PMC56910

[15] Punkvang A, Saparpakorn P, Hannongbua S, Wolschann P, Beyer A, et al (2010) Investigating the structural basis of arylamides to improve potency against *M. tuberculosis* strain through molecular dynamics simulations. Eur J Med Chem 45: 5585–5593. 20888672 10.1016/j.ejmech.2010.09.008

[16] Schaffner-Barbero C, Gil-Redondo R, Ruiz-Avila LB, Huecas S, Lappchen T, et al (2010) Insights into nucleotide recognition by cell division protein FtsZ from a mant-GTP competition assay and molecular dynamics. Biochemistry 49: 10458–10472. 21058659 10.1021/bi101577p

[17] Hu YJ, Liu Y, Zhang LX (2005) Studies of interaction between colchicine and bovine serum albumin by fluorescence quenching method. J Mol Struct 750: 174–176.

[18] Lakowicz JR, Weber G (1973) Quenching of fluorescence by oxygen. A probe for structural fluctuations in macromolecules. Biochemistry 12: 4161–4170. 4795686 10.1021/bi00745a020 PMC6959846

[19] Ragle BE, Bubeck Wardenburg J (2009) Anti-alpha-hemolysin monoclonal antibodies mediate protection against *Staphylococcus aureus* pneumonia. Infect Immun 77: 2712–2718. 19380475 10.1128/IAI.00115-09 PMC2708543

[20] Iwase T, Uehara Y, Shinji H, Tajima A, Seo H, et al (2010) *Staphylococcus epidermidis* Esp inhibits *Staphylococcus aureus* biofilm formation and nasal colonization. Nature 465: 346–349. 20485435 10.1038/nature09074

[21] Rasko DA, Sperandio V (2010) Anti-virulence strategies to combat bacteria-mediated disease. Nat Rev Drug Discov 9: 117–128. 20081869 10.1038/nrd3013

[22] McCormick CC, Caballero AR, Balzli CL, Tang A, O'Callaghan RJ (2009) Chemical inhibition of alpha-toxin, a key corneal virulence factor of *Staphylococcus aureus* . Invest Ophthalmol Vis Sci 50: 2848–2854. 19136695 10.1167/iovs.08-3157

[23] Reddy NR, Erion MD (2001) Calculation of relative binding free energy differences for fructose 1, 6-bisphosphatase inhibitors using the thermodynamic cycle perturbation approach. J Am Chem Soc 123: 6246–6252. 11427047 10.1021/ja0103288

[24] Zhang WF, Sulea T, Tao LM, Cui QZ, Purisima EO, et al (2011) Contribution of Active Site Residues to Substrate Hydrolysis by USP2: Insights into Catalysis by Ubiquitin Specific Proteases. Biochemistry 50: 4775–4785. 21542621 10.1021/bi101958h

[25] Chong TL, Duan Y, Wang L, Massova I, Kollman PA (1999) Molecular dynamics and free-energy calculations applied to affinity maturation in antibody 48G7. Proc Natl Acad Sci USA 96: 14330–14335. 10588705 10.1073/pnas.96.25.14330 PMC24436

[26] Souza PCT, Barra GB, Velasco LFR, Ribeiro Isabel CJ, Simeoni LA, et al (2011) Helix 12 Dynamics and Thyroid Hormone Receptor Activity: Experimental and Molecular Dynamics Studies of Ile280 Mutants. J Mol Biol 412: 882–893. 21530542 10.1016/j.jmb.2011.04.014

[27] Filomia F, Rienzo FD, Menziani MC (2010) Insights into MAPK p38a DFG flip mechanism by accelerated molecular dynamics. Bioogranic & Medicinal Chemistry 18: 6805–6812. 10.1016/j.bmc.2010.07.047 20724167

[28] Wang J, Morin P, Wang W, Kollman PA (2001) Use of MM-PBSA in reproducing the binding free energies to HIV-1 RT of TIBO derivatives and predicting the binding mode to HIV-1 RT of Efavirenz by docking and MM-PBSA. J Am Chem Soc 123: 5221–5230. 11457384 10.1021/ja003834q

[29] Meher Biswa Ranjan, Wang Yixuan (2012) Interaction of I50V Mutant and I50L/A71V Double Mutant HIV-Protease with Inhibitor TMC114 (Darunavir): Molecular Dynamics Simulation and Binding Free Energy Studies. J Phys Chem B 116: 1884–1900. 22239286 10.1021/jp2074804 PMC3288396

[30] Istyastono EP, Nijmeijer S, Lim HD, Stolpe Avan de, Roumen L, et al (2011) Molecular Determinants of Ligand Binding Modes in the Histamine H_4_ Receptor: Linking Ligand-Based Three-Dimensional Quantitative Structure–Activity Relationship (3D-QSAR) Models to in Silico Guided Receptor Mutagenesis Studies. J Med Chem 54: 8136–8147. 22003888 10.1021/jm201042n

[31] Peng C, Gordon TH (2011) The dynamical mechanism of auto-inhibition of AMP-activated protein Kinase. PLoS Computational Biology 7: e1002082. 21814500 10.1371/journal.pcbi.1002082 PMC3140967

[32] Kaushik S, Sowdhamini R (2011) Structural Analysis of Prolyl Oligopeptidases Using Molecular Docking and Dynamics: Insights into Conformational Changes and Ligand Binding. PLoS ONE 6: e26251. 22132071 10.1371/journal.pone.0026251 PMC3223163

[33] Basse N, Kaar JL, Settanni G, Joerger AC, Rutherford TJ, et al (2010) Toward the rational design of p53-stabilizing drug: probing the surface of the oncogenic Y220C mutant. Chemistry & Biology Cell press 17: 46–56. 10.1016/j.chembiol.2009.12.011 20142040

[34] Lawrence S, Hackett JC, Moran RG (2011) Tetrahydrofolate Recognition by the Mitochondrial Folate Transporter. J Biol Chem 286: 31480–31489. 21768094 10.1074/jbc.M111.272187 PMC3173085

[35] Perryman A, Forli S, Morris GM, Burt C, Cheng YH, et al (2010) A Dynamic Model of HIV Integrase Inhibition and Drug Resistance. J Mol Biol 397: 600–615. 20096702 10.1016/j.jmb.2010.01.033 PMC2846753

[36] Savatier ND, Poggi L, Miclet E, Lopes P, Ouazzani J, et al (2009) Insight Into the Enzymatic Mechanism of 6-Phosphogluconolactonase from *Trypanosoma brucei* Using Structural Data and Molecular Dynamics Simulation. J Mol Biol 388: 1009–1021. 19345229 10.1016/j.jmb.2009.03.063

[37] Qiu J, Zhang X, Luo M, Li H, Dong J, et al (2011) Subinhibitory concentrations of perilla oil affect the expression of secreted virulence factor genes in *Staphylococcus aureus* . PLoS One 6: e16160. 21283822 10.1371/journal.pone.0016160 PMC3023776

[38] Bharatam PV, Khanna S, Francis SM (2008) Modeling and informatics in drug design. In: Cox GAD, editor. *Preclinical Development Handbook: ADME and Biopharmaceutical Properties*. Hoboken, NJ: John Wiley & Sons, Inc. pp. 1–45.

[39] Fiser A, Do RK, Sali A (2000) Modeling of loops in protein structures. Protein Sci 9: 1753–1773. 11045621 10.1110/ps.9.9.1753 PMC2144714

[40] Hess B, Kutzner C, Van der Spoel D, Lindahl E (2008) GROMACS 4: algorithms for highly efficient, load-balanced, and scalable molecular simulation. J Chem Theory Comput 4: 435–447. 26620784 10.1021/ct700301q

[41] Frisch MJ, Trucks GW, Schlegel HB, Scuseria GE, Robb MA, et al. (2009) Gaussian 09, Wallingford, CT, Gaussian 09 Revision A.02. Wallingford, CT: Gaussian Inc.

[42] Morris GM, Huey R, Lindstrom W, Sanner MF, Belew RK, et al (2009) AutoDock4 and AutoDockTools4: Automated docking with selective receptor flexibility. J Comput Chem 30: 2785–2791. 19399780 10.1002/jcc.21256 PMC2760638

[43] Hu R, Barbault F, Maurel F, Delamar M, Zhang R (2010) Molecular dynamics simulations of 2-amino-6-arylsulphonylbenzonitriles analogues as HIV inhibitors: interaction modes and binding free energies. Chem Biol Drug Des 76: 518–526. 20942836 10.1111/j.1747-0285.2010.01028.x

[44] Morris GM, Goodsell DS, Huey R, Olson AJ (1996) Distributed automated docking of flexible ligands to proteins: parallel applications of AutoDock 2.4. J comput-aided mol des 10: 293–304. 8877701 10.1007/BF00124499

[45] Hu R, Barbault F, Delamar M, Zhang R (2009) Receptor- and ligand-based 3D-QSAR study for a series of non-nucleoside HIV-1 reverse transcriptase inhibitors. Bioorg Med Chem 17: 2400–2409. 19250835 10.1016/j.bmc.2009.02.003

[46] Jorgensen WL, Chandrasekhar J, Madura JD, Impey RW, Klein ML (1983) Comparison of simple potential functions for simulating liquid water. J Chem Phys 79: 926–935.

[47] Ryckaert JP (1997) Numerical integration of cartesian equations of motion of a system with constrained molecular dynamics of N-alkanes. J Chem Phys 23: 327–341.

[48] Berendsen HJC, Postma JPM, van Gunsteren WF, Dinola A, Haak JR (1984) Molecular dynamics with coupling to an external bath. J Chem Phys 81: 3684–3690.

[49] Wang J, Wang W, Kollman PA, Case DA (2006) Automatic atom type and bond type perception in molecular mechanical calculations. J Mol Graph Model 2: 247–260. 10.1016/j.jmgm.2005.12.005 16458552

[50] Jakalian A, Jack DB, Bayly CI (2002) Fast, efficient generation of high-quality atomic charges. AM1-BCC model: II. Parameterization and validation. J Comput Chem 23: 1623–1641. 12395429 10.1002/jcc.10128

[51] Ibrahim N, Ibrahim H, Kim S, Nallet JP, Nepveu F (2010) Interactions between antimalarial indolone-*N*-oxide derivatives and human serum albumin. Biomacromolecules 11: 3341–3351. 21080702 10.1021/bm100814n

[52] Jurasekova Z, Marconi G, Sanchez-Cortes S, Torreggiani A (2009) Spectroscopic and molecular modeling studies on the binding of the flavonoid luteolin and human serum albumin. Biopolymers 91: 917–927. 19603495 10.1002/bip.21278

[53] Bandyopadhyay S, Valder CR, Huynh HG, Ren H, Allison WS (2002) The βG^156^C substitution in the F_1_-ATPase from the thermophilic *Bacillus* PS3 affects catalytic site cooperativity by destabilizing the closed conformation of the catalytic site. Biochemistry 41: 14421–14429. 12450409 10.1021/bi026243g

[54] Bhakdi S, Fussle R, Tranum-Jensen J (1981) Staphylococcal alpha-toxin: oligomerization of hydrophilic monomers to form amphiphilic hexamers induced through contact with deoxycholate detergent micelles. Proc Natl Acad Sci U S A 78: 5475–5479. 6272304 10.1073/pnas.78.9.5475 PMC348768

[55] Qiu J, Feng H, Xiang H, Wang D, Xia L, et al (2010) Influence of subinhibitory concentrations of licochalcone A on the secretion of enterotoxins A and B by *Staphylococcus aureus* . FEMS Microbiol Lett 307: 135–141. 20412304 10.1111/j.1574-6968.2010.01973.x

